# Quality assurance: A document review of moderation reports on final theoretical assessments

**DOI:** 10.4102/hsag.v30i0.3024

**Published:** 2025-08-20

**Authors:** Gabieba Donough, Katlego Mthimunye, Felicity Daniels

**Affiliations:** 1 Faculty of Community and Health Sciences, University of the Western Cape, Cape Town, South Africa

**Keywords:** assessment design, external moderation, final theoretical assessment, internal moderation, nursing education, quality assurance

## Abstract

**Background:**

Ensuring assessment quality in nursing education is crucial for preparing graduates for healthcare demands, particularly in South Africa, where healthcare systems face challenges. Moderation serves as a quality assurance tool, yet its effectiveness remains underexplored.

**Aim:**

To review moderation reports on final theoretical assessments of nursing modules in the R425 Bachelor of Nursing programme.

**Setting:**

A higher education institution (HEI) in the Western Cape, South Africa.

**Methods:**

A qualitative exploratory descriptive study analysed 70 moderation reports (2015–2019) using a structured data extraction tool and content analysis.

**Results:**

One main theme emerged, quality assurance in assessment design, with five categories: standard of questions and alignment with Bloom’s Taxonomy and NQF levels; linguistic presentation and clarity of questions; clarity of instructions to students; duration and mark allocation; and attainment of module outcomes.

**Conclusion:**

The study revealed persistent gaps in assessment quality, including misalignment with cognitive levels, unclear instructions, inconsistent mark allocation, and limited outcome alignment. These findings reinforce the need for robust moderation processes to support fair, valid, and reliable assessments. Recommendations include strengthening assessor training, implementing structured moderation frameworks, and improving documentation.

**Contribution:**

This study highlights the critical role of quality assurance in final theoretical assessments by identifying recurring issues in assessment design and moderation. It offers practical insights to enhance assessment integrity and ensure alignment with educational standards.

## Introduction and background

The moderation of assessments plays a crucial role in quality assurance, ensuring that assessments are fair, valid and reliable, and that they align with the intended learning outcomes of a programme (Hecker et al. 2024; Williams, Johnston & Averill 2023). Higher education institutions (HEIs) have a responsibility to maintain high academic standards and moderation serves as a safeguard to ensure that assessment practices adhere to these standards (Mitchell, Hara & Sheriff 2019; Paterson et al. 2020). This is particularly important in nursing education, where the outcomes of theoretical assessments directly impact clinical competence, patient safety and professional readiness (Erlinger 2018; South African Nursing Council [SANC] 2021a). Because of the nature of their profession, nursing graduates must demonstrate not only theoretical knowledge but also the critical thinking abilities required to provide safe and effective patient care in pressurised environments.

In South Africa, assessment design is regulated by the South African Qualifications Authority (SAQA) and structured according to the National Qualifications Framework (NQF) (Mokwele & Chetty 2022). The NQF standardises educational qualifications, ensuring consistency, quality and alignment with global academic and professional benchmarks (SAQA 2022). The Bachelor of Nursing (BN) degree, on which the study is based, spans various NQF levels: NQF Level 5 (ECP and BN1), NQF Level 6 (BN2), NQF Level 7 (BN3) and culminates in NQF Level 8 (BN4). Assessments are scaffolded from lower-order cognitive skills such as remembering, understanding and applying to higher-order cognitive skills such as analysis, evaluation and creation (Mokwele & Chetty 2022; SAQA 2022). Higher education institutions must ensure that assessments align with NQF level descriptors, which define the expected cognitive complexity and learning outcomes for each qualification level (Meyer 2019; SAQA 2022). Moderation is a key mechanism for ensuring that assessments meet these national and institutional quality assurance standards (Williams et al. 2023).

Despite the structured assessment regulations, inconsistencies in assessment design and moderation have been reported (Dos Reis et al. 2022; Fayilane 2017). The SANC, as the Education and Training Quality Assurance (ETQA) body, mandates that nurse educators be registered as assessors and moderators (SANC 2021b). However, no additional training is required for these roles, which may lead to variability in assessment quality and moderation practices. Research indicates that assessments often fail to consistently align with the expected NQF level descriptors and a taxonomy, raising concerns about the validity and cognitive challenge of the questions posed (Dos Reis et al. 2022; Hecker et al. 2024; Masuku, Jili & Sabela 2021). Therefore, this study sought to explore the extent to which quality assurance practices are implemented in the design and moderation of final theoretical assessments within a nursing education institution. The study is grounded in the author’s original thesis, which served as the foundation for this study. The document review of moderation reports was one of several studies conducted as part of a larger study to examine assessment design practices, alongside a scoping review, student survey and educator interviews.

Given the critical role of moderation in safeguarding assessment validity and reliability (Williams et al. 2023), it is essential to explore how effectively these processes uphold quality assurance standards in nursing education. Moderation functions as a formal mechanism for maintaining alignment with NQF levels and cognitive taxonomies (Williams et al. 2023), yet variations in implementation raise questions about its effectiveness as a quality assurance tool. This study expands on existing moderation report data by systematically analysing how theoretical assessments reflect cognitive rigour, fairness and alignment with academic expectations. By exploring moderation reports, the study offers a structured evaluation of assessment design and moderation practices within a regulated higher education framework. This contributes to a deeper understanding of how quality assurance mechanisms promote assessment integrity, consistency and alignment with institutional and national standards in nursing education.

### Problem statement

Inconsistencies in assessment design and moderation practices, including misalignment with cognitive level descriptors and unclear instructions, compromise the validity and reliability of summative assessments. These assessments are critical for assessing students’ knowledge, competency and readiness for professional practice or progression to the next NQF level (Donough, Daniels & Mthimunye 2022; Dos Reis et al. 2022; Masuku et al. 2021). Assessments must be free from ambiguity, appropriately aligned with module outcomes and structured to ensure reliable student performance assessment (Boud & Bearman 2024). Despite their importance, research on the implementation and effectiveness of moderation practices in nursing education is limited.

Previous studies have highlighted the prevalence of lower-order cognitive skills in assessments and poor alignment with module outcomes, reinforcing the need for robust moderation processes to safeguard assessment quality (Dos Reis et al. 2022; Fayilane 2017; Meyer 2019). Research suggests that moderation reports are often underutilised, with limited institutional reflection on the trends, challenges and inconsistencies in assessment design (Alemu 2024; Masuku et al. 2021; Meyer 2019). Despite its role in ensuring validity and fairness, moderation is often overlooked (Beutel, Adie & Lloyd 2017). Some studies highlighted misalignment between taught content, assessments and graduate expectations, further reinforcing the need for strong quality assurance mechanisms in assessment design and moderation (Dos Reis et al. 2022; Fayilane 2017). This study addresses these gaps by reviewing both the internal and external moderation reports from a South African nursing programme to evaluate the quality assurance of final theoretical assessments.

### Theoretical framework

This study is guided by Bloom’s Taxonomy (Bloom 1956) and Biggs’ (Biggs 1996) Constructive Alignment framework. Bloom’s Taxonomy offers a hierarchical structure for classifying cognitive skills, from lower-order (e.g. remembering) to higher-order thinking (e.g. creating), ensuring assessments align with learning outcomes and challenge students appropriately. In nursing education, this is critical for developing both foundational knowledge and clinical reasoning skills essential for safe patient care (Donough 2023; Wong & Kowitlawakul 2020). Additionally, the Biggs’ Constructive Alignment emphasises aligning teaching strategies, learning activities and assessments with intended outcomes. Misalignment risks superficial assessments, leading to competency gaps (Fayilane, 2017; Hecker et al. 2024; Masuku et al. 2021). This framework ensures assessments accurately measure student achievement against programme goals. By integrating these frameworks, the study analyses moderated assessments in a South African nursing programme for validity, reliability, fairness, alignment and cognitive challenge, offering insights to enhance quality assurance in nursing education assessments.

### Aim and rationale

This study aims to review moderation reports on final theoretical assessments of nursing modules in the R425 Bachelor of Nursing programme at a HEI in the Western Cape, South Africa. By exploring what is reported by moderators, the study can identify areas for improvement to enhance the quality of assessments and provide evidence-based insights to strengthen theoretical assessment design in nursing education.

## Research methods and design

### Research design

This study employed a qualitative exploratory descriptive design (Creswell & Creswell 2017) to explore both the internal and external moderation reports from the Regulation 425 (R425) undergraduate nursing programme at a selected HEI in South Africa. The qualitative document review aimed to identify trends, gaps and recommendations (Creswell & Creswell 2017) within the moderation feedback related to final theoretical assessments. The R425 programme, which included a 4-year Bachelor of Nursing and a 5-year Extended Curriculum Programme (ECP), was phased out nationally in 2019 and replaced by the R174 Bachelor of Nursing programme in January 2020 (SANC 2021a). The findings and recommendations derived from this analysis may offer valuable insights for the new R174 programme.

### Research setting

This study was conducted within a nursing school at a HEI in the Western Cape, South Africa, which offers both undergraduate and postgraduate nursing programmes. Data collection focused on moderation reports from the R425 undergraduate nursing programme over a 5-year period (2015–2019). This timeframe was selected to capture assessment practices in the final years of the R425 programme, allowing for a longitudinal review of moderation feedback leading up to its national phase-out in 2019. The selection aligns with pre–post-transition analysis principles, enabling a comprehensive assessment of existing practices before the introduction of the R174 programme in 2020 (SANC 2021a). The findings provide insights that may inform assessment design within the new programme.

### Sampling

A total of 150 internal and external moderation reports for 18 nursing modules were initially obtained from institutional archives and departmental records, managed by the undergraduate administrator responsible for filing these reports. These reports were available in both electronic and hard copy formats. Inclusion criteria required that reports be complete, pertain to final theoretical assessments and provide feedback on nursing module assessments from the period 2015–2019. Reports related to non-nursing modules, postgraduate modules and clinical modules were excluded to maintain focus. Additionally, 80 reports were excluded due to incomplete documentation or poor record-keeping, as such reports could compromise the reliability and validity of the analysis. This exclusion ensured that the remaining data adhered to the criteria necessary for a thorough and trustworthy analysis. To ensure comprehensive coverage and thematic saturation, a purposive sampling method was employed, resulting in the selection of 70 reports (22 internal and 48 external reports). This sample encompassed theoretical assessments across different years and academic levels, providing a representative overview of theoretical assessment practices within the undergraduate nursing programme.

### Data extraction and analysis

A data extraction tool was developed based on the university’s moderation report template to systematically collect relevant information. The tool was pre-tested and validated for content and face validity, ensuring its effectiveness (Haq et al. 2023). Data were extracted from both hard and electronic copies of the reports. Content analysis, following Krippendorff’s (2018) five-step framework, was used to analyse the data: (1) Relevant content within the moderation reports was identified and segmented into smaller, meaningful units to facilitate analysis. (2) Reports that met the inclusion criteria were selected to ensure the data were relevant, comprehensive and representative of the study’s aim. (3) The extracted data were categorised into meaningful units based on emerging themes and patterns related to assessment quality and moderation feedback. (4) The coded data were integrated and analysed to identify recurring trends, inconsistencies and key findings in assessment design. (5) Conclusions were drawn based on the synthesised data, providing interpretations and recommendations to improve quality assurance in theoretical assessment design.

An iterative process was followed, allowing findings from the data extraction to inform and refine subsequent analyses (Harrison, Reilly & Creswell 2020). This approach ensured a thorough understanding of the moderators’ feedback and its implications for assessment design. The systematic and iterative nature of the analysis provided robust insights into the strengths and areas for improvement in the design of final theoretical assessments during the specified period.

### Measures of trustworthiness

This study adhered to established trustworthiness criteria to ensure rigour and integrity in its findings. Credibility was ensured by including only completed moderation reports, guaranteeing the accuracy and reliability of the data. Transferability was achieved through detailed descriptions of the research context, methodology and findings, allowing readers to assess the relevance and applicability of the study to similar educational settings. Dependability was maintained through a structured and systematic research process, ensuring consistency and replicability, with clear documentation of procedures reinforcing reliability. Confirmability was strengthened by implementing strategies to minimise bias, including bracketing researcher assumptions, pre-testing the data extraction tool for validation and maintaining an audit trail for transparency. By integrating these methodological safeguards, the study upholds the principles of trustworthiness, ensuring its conclusions are well-founded and applicable to nursing education assessment practices.

### Ethical considerations

Ethical approval was obtained from the Human Social Sciences Research Ethics Committee at the institution (Reference: HS20/8/19). Permission to review moderation reports was granted by the registrar and the director of the selected school of nursing included in the study. To ensure confidentiality and data protection, all identifying information, including moderator names, module names and institutional details, was anonymised. Electronic data, including transcripts, were stored on password-protected, encrypted devices accessible only to the researcher. In line with institutional and ethical guidelines, data will be retained for 5 years before secure disposal (Creswell & Creswell 2017).

The researcher, who was a staff member at the selected nursing school, has taken measures to ensure an objective and unbiased analysis (Sims 2020). The researcher employed bracketing techniques to set aside personal biases and preconceptions with data collection and analysis (Creswell & Creswell 2017). These measures upheld ethical standards, safeguarding privacy throughout the research process and upholding trustworthiness in the research process.

## Results

One main theme emerged: Quality assurance in assessment design with five key categories. Table 1 summarises the theme and its categories:

**TABLE 1:** Themes and categories.

Main theme	Categories
Quality assurance in assessment design	– Standard of questions and alignment with Bloom’s Taxonomy and NQF levels – Linguistic presentation and clarity of questions – Clarity of instructions to students – Duration of examination and mark allocation – Attainment of module outcomes

NQF, national qualifications framework.

### Quality assurance in assessment design

Quality assurance in assessment design is essential to maintaining the validity, reliability and fairness of assessments in nursing education (Oermann, Gaberson & De Gagne 2024). It ensures alignment with learning outcomes, appropriate cognitive levels and clarity in question formulation (Boud & Bearman 2024). The findings highlight key concerns related to quality assurance, including inconsistencies in question standardisation, linguistic precision, instructional clarity, mark distribution and outcome alignment. The quotations are moderator comments extracted from the moderation reports.

**Standard of questions and alignment with Bloom’s Taxonomy and national qualifications framework levels:** The findings revealed inconsistent alignment between assessment questions, Bloom’s Taxonomy and NQF levels. Many moderation reports noted that questions primarily tested lower-order cognitive skills such as recall and comprehension, with limited emphasis on higher-order thinking:

‘Bloom’s Taxonomy for knowledge, application and comprehension mostly tested, therefore NQF level 6 not applied throughout the three examination papers.’ (2nd Year, External Moderator, Moderator A)

‘Level of questions is primarily based on “understanding”.’ (3rd Year, External Moderator, Moderator A)

‘Suggestions that the formulation of MCQs be at higher levels of knowledge.’ (3rd Year, External Moderator, Moderator B)

These comments indicate a tendency to focus on lower-order questions, with limited opportunities for students to demonstrate analytical and critical thinking skills.

**Linguistic presentation and clarity of questions:** Moderation reports highlighted common grammatical errors, ambiguous wording and double-barrelled questions that could affect students’ understanding:

‘Some questions have two questions in one. The students might miss the other question and lose marks.’ (3rd Year, External Moderator, Moderator C)

‘Do not use double-barrel questions.’ (3rd Year, External Moderator, Moderator D)

‘Grammar and spelling need attention.’ (3rd Year, External Moderator, Moderator E)

‘The sentence structure and grammar used in scenarios may confuse students and affect clarity.’ (2nd Year, Internal Moderator, Internal Moderator F)

These comments suggest that unclear and inconsistent question phrasing is a recurring concern.

**Clarity of instructions to students:** The findings indicate that unclear or incomplete instructions in assessments were a common issue, which can hinder students’ understanding and performance:

‘Instruction to students is not clear for all three papers.’ (ECP/1st Year, Internal Moderator, Moderator B)

‘There are some suggestions I made in terms of clarity of instructions and questions.’ (ECP/1st Year, Internal Moderator, Moderator C)

Ensuring clarity in assessment instructions is important for students’ understanding and performance. The moderation comments highlight concerns regarding unclear instructions, suggesting a need for structured guidelines to improve clarity, transparency and fairness in assessment.

**Duration of examination and mark allocation:** Moderation reports revealed inconsistencies in the allocation of marks and concerns regarding examination duration:

‘Concerned about the [*exam*] time frame – two hours for 50 marks.’ (2nd Year, External Moderator, Moderator B)

‘There are no allocated marks for these questions.’ (ECP/1st Year, Internal Moderator, Moderator D)

‘Question 2 is misleading to students. Question paper indicates 5 × 1 = 5, memo indicates 10 × ½ = 5.’ (4th Year, External Moderator, Moderator A)

These findings suggest that discrepancies in mark allocation and examination duration could impact the fairness and consistency of assessments.

**Attainment of module outcomes:** The moderation reports indicated gaps in aligning assessments with module outcomes, with several moderators noting that not all intended learning outcomes were sufficiently addressed:

‘Each paper lacked assessment of all specific outcomes for this module.’ (2nd Year, Internal Moderator, Moderator C)

‘No indication of the learning outcomes. Learning outcomes were either insufficiently assessed or overlooked.’ (3rd Year, External Moderator, Moderator F)

These findings point toward concerns about the completeness of assessment coverage and alignment with expected competencies.

## Discussion

This study reviewed moderation reports on final theoretical assessments of a nursing programme at a HEI. The findings of this study revealed quality assurance gaps that impact the validity, reliability and fairness of assessments. These gaps include inconsistent alignment with cognitive frameworks, linguistic errors, unclear instructions, discrepancies in mark allocation and incomplete coverage of learning outcomes. These findings highlight the need for structured quality assurance processes to ensure that theoretical assessments promote deep learning and support students’ preparedness for professional nursing practice.

The findings of this study indicate inconsistent alignment of assessment questions with Bloom’s Taxonomy and NQF levels. Many questions predominantly assessed lower-order cognitive skills such as recall and comprehension, while neglecting higher-order reasoning and critical thinking. Fayilane (2017), in a study analysing the cognitive levels of final examination questions for a nursing programme in the Eastern Cape, similarly found that assessments were predominantly pitched at lower-order thinking skills, with limited focus on higher-order cognitive skills and alignment with learning outcomes. Furthermore, Bearman et al. (2016) observed that while educators often believe they know how to design quality assessments, the reality revealed misalignment and inadequate scaffolding of higher-order thinking skills are common. This misalignment suggests educators require targeted training in assessment question design to ensure students are challenged appropriately (Kotcherlakota et al. 2024). Strengthening educators’ capacity to design assessments that promote critical thinking is essential for preparing nursing students for the complex demands of professional practice.

Linguistic issues emerged as a significant concern, with moderation reports citing grammatical errors, ambiguous wording and double-barrelled questions, which could cause students to misinterpret questions and lose marks. Paterson et al. (2020) studied on ‘What are students’ needs and preferences in higher education’ and found that unclear language in assessments can lead to misinterpretation, negatively impacting academic performance and fairness in grading. This study was supported by Ergashevich (2023) and Irenka and Ireland (2021), who advocated for technical writing training for educators and integrating linguistic review into moderation processes to enhance clarity. Improving technical writing and linguistic precision in assessments reduces ambiguity, enhances fairness and ensures students can accurately interpret questions, ultimately supporting valid assessment outcomes. Such measures are essential to improving assessment principles and fostering a fair and equitable learning environment.

Furthermore, the findings of this study indicate that unclear and inconsistent assessment instructions are a recurring issue, negatively affecting students’ understanding and performance. Villarroel et al. (2024) and Ajjawi et al. (2024) argued that clear, well-structured instructions are a fundamental principle of fair assessment practices. Lack of clarity in instructions may hinder students’ ability to respond accurately. Ambiguous instructions risk student misinterpretation, leading to incorrect responses and unfair grading. Hence, standardised, explicit and accessible instructions help establish a shared understanding between students and educators. Educators should ensure that the instructional phrasing is clear, direct and universally understood, minimising the risk of student misinterpretation. Establishing standardised guidelines for instructions can improve transparency, consistency and fairness in assessments.

Concerns related to examination duration and mark allocation were highlighted in the moderation reports. These discrepancies alluded to the need for standardised timing guidelines and transparent grading rubrics to support fairness and reliability. This finding aligns with Cao et al. (2019), who emphasised the importance of using standardised timing guidelines and clear marking rubrics. Benchmarking examination duration against established timing guidelines, such as the 91.5 s per question rule, can help ensure that students have adequate time to complete assessments (Mitchell et al. 2019; SAFe 2021). Similarly, using well-defined rubrics for mark allocation enhances consistency and ensures that grading reflects the cognitive demands of each question. Establishing alignment between time allocation and question complexity, along with consistent application of rubrics, is essential to minimise disparities and promote equitable assessment practices.

The moderation reports further revealed gaps in constructive alignment between assessments and module outcomes. Several moderation reports indicated that examination papers lacked alignment with module outcomes raising concerns about incomplete measurement of student knowledge and competencies. This aligns with research by Hecker et al. (2024), who emphasised the risk of inadequate assessment when examinations are not properly mapped to learning outcomes. Boud and Bearman (2024) affirmed that quality assurance mechanisms in assessment design required structured moderation processes, enhanced training for assessors and clear institutional guidelines. Strengthening constructive alignment through curriculum mapping and moderation practices is essential to ensure that theoretical assessments accurately measure students’ knowledge, skills and readiness for professional practice.

While HEIs are required to implement quality assurance measures, this study demonstrates that merely having policies and procedures in place is insufficient. Ongoing investment in building assessment literacy among educators, refining moderation practices and ensuring alignment with cognitive frameworks and learning outcomes is critical for enhancing the integrity of nursing education assessments. By integrating the study’s findings into future moderation and assessment design processes, nursing programmes can enhance the quality, fairness and validity of their theoretical assessments, ultimately supporting the development of competent, professionally prepared nursing graduates equipped to meet the complex demands of the profession.

### Limitations

This study was conducted at a single HEI and focused specifically on moderation reports from the R425 Bachelor of Nursing programme, making the findings context-specific. Additionally, some moderation reports were incomplete or lacked detailed commentary, which may have limited the depth and comprehensiveness of the analysis.

### Recommendations

#### For nursing education

- Provide ongoing training for assessors and moderators to strengthen their skills in assessment design, ensuring constructive alignment with NQF level descriptors and a taxonomy. Regular evaluations of these training outcomes should be conducted to ensure proper implementation and lasting improvement.
- Conduct regular workshops focused on quality assurance practices in assessment. These workshops should cover question design, adherence to moderation guidelines and effective documentation processes. Include practical exercises and case studies to encourage hands-on learning and application.

#### For future research

- Conduct similar reviews of moderation reports in other nursing education institutions to benchmark current practices, identify areas for improvement and ensure ongoing refinement in assessment quality.

These recommendations complement existing quality assurance practices by addressing persistent gaps through targeted enhancements. Implementing these strategies will improve assessment integrity, fairness and alignment with educational standards. Furthermore, these actions will strengthen documentation processes, refine moderation practices and ensure the continuous development and capacity building of assessors and moderators.

## Conclusion

This study provided valuable insights into quality assurance in the design of final theoretical assessments in nursing education by analysing both the internal and external moderation reports. While quality assurance processes and training programmes exist within institutions, the findings highlight persistent gaps and inconsistencies in their application. This study complements existing mechanisms by recommending targeted enhancements to address these challenges.

Key issues identified include misalignment of assessments with a taxonomy and NQF levels, lack of clarity in question phrasing and instructions, discrepancies in examination duration and mark allocation and gaps in aligning assessments with module outcomes. Significantly, many theoretical assessments primarily tested lower-order cognitive skills, failing to sufficiently challenge students at the appropriate NQF level.

These findings reinforce the need for ongoing improvements in assessment design and moderation processes. Strengthening alignment with a cognitive taxonomy and NQF levels, ensuring linguistic clarity and providing targeted training for assessors and moderators are critical for promoting fairness, validity and the development of higher-order thinking and skills. Establishing clear examination duration and mark allocation benchmarks further support assessment integrity. While this study focused on nursing education, its findings are relevant to other higher education disciplines that rely on moderation for assessment quality assurance. By strengthening assessment design, documentation and moderation processes, HEIs can improve assessment integrity, fairness and alignment with NQF levels, ultimately supporting student learning, competency development and professional readiness.
